# Recombination-mediated gene-specific sweeps in motion across the human gut microbiome

**DOI:** 10.20517/mrr.2026.32

**Published:** 2026-09-23

**Authors:** Matthew Foley, Rodolphe Barrangou

**Affiliations:** Department of Food, Bioprocessing and Nutrition Sciences, North Carolina State University, Raleigh, NC 27606, USA.

The human gut microbiome symbiotically and opportunistically co-evolved with its host over millions of years of our speciation resulting in a diverse array of commensal strains well-adapted to their human hosts over that timeframe^[1,2]^. While evolution can occur and scale broadly at the population level, commensal strains are capable of displaying singular adaptive evolution within individual microbiomes, illustrating the efficiency and resolution of natural selection. Bacterial genomic plasticity is driven by mutation, genetic drift, and recombination-mediated horizontal gene transfer (HGT) that results in the stable introduction of new genes. The permits the microbiome’s adaptation in response to new environmental pressures, outpacing our own evolution^[3]^. In particular, HGT is thought to be a foundational mechanism of bacterial adaptation, supporting the evolution of commensal strains that display new functionality reflective of host lifestyle and enable genome expansion through the acquisition of beneficial genetic material from the environmental context. HGT can similarly result in the spread of neutral or deleterious mutations via non-selective forces that confound the inference of adaptive and non-adaptive alleles in genomic analyses. The rise of industrialized and urban populations in recent human history has likely further selected for swift bacterial adaptation, as evidenced by increased occurrence of HGT in these populations^[4]^. However, the efficiency by which HGT results in microbiome evolution, and a systematic accounting of which bacterial gene functions support adaptation, remains unclear. 

Recently, Wolff and Garud address this gap using a statistical approach to detect adaptive genes spreading through microbiomes by HGT^[5]^. By implementing a new population-genetics framework to detect recombination-mediated selective sweeps (adaptive variants that rapidly rise to high frequency in a population), the authors show that individual genes harboring specific functions, not necessarily whole genomes, are repeatedly spread and enriched through gut bacterial populations worldwide. This analysis was carried out through the development of an integrated linkage disequilibrium score (iLDS), a statistic specifically designed to scan bacterial genomes for signatures of positive selection of genetic variants. Classical approaches for detecting positive selection are largely formulated in sexually reproducing eukaryotes or in clonal microbial systems, where beneficial mutations often rise through genome-wide selective sweeps. However, HGT enables adaptive loci to move across bacterial strains independent of the chromosome. Subsequently, adaptive evolution can proceed through gene-specific rather than genome-wide sweeps. By exploiting local patterns of linkage disequilibrium among synonymous and non-synonymous variants, the authors were able to infer recent examples of microbiome adaptive evolution across human populations and geographies.

When applied to 30 of the most prevalent gut commensals or more broadly to large metagenomic datasets from two dozen global populations, this methodology identified 155 and 309 candidate selective sweeps, respectively, with multiple events sometimes detected per species, reflecting a broad and consistent adaptive pattern. Many sweeps were geographically restricted, consistent with local adaptation, but a minority were shared across continents, reflecting local effects of a broad phenomenon. Previous studies have documented rapid within-host adaptation over days to months, often following ecological perturbation^[3]^. By contrast, the present study demonstrated that adaptive loci can also spread between hosts and across populations, implying that the gut microbiome contains a globally connected reservoir of beneficial genetic variation subject to recurrent selection. Notably, genes involved in carbohydrate acquisition, transport, and catabolism were significantly enriched among sweeps, reflecting the importance of nutrient scavenging. Hallmark carbohydrate importers from polysaccharide utilization loci (PULs) were repeatedly identified^[6]^, suggesting that the targeting of specific, recently introduced carbohydrates is providing the selective pressure that mediates adaptation. Contextually, this also suggests that eating habits in the human host population are drivers of gut microbiome evolution, and that shifts in human diet patterns would drive related changes in gut bacterial genomes.

In particular, the *Ruminococcus *and *Eubacterium*-encoded *mdxEF* locus, involved in maltodextrin utilization, was observed and highlighted as being under strong selection by iLDS^[5,7]^. In the species *Ruminococcus bromii*, this selection was associated with geographical origin; *mdxEF *sweeps were identified across industrialized populations yet were absent from surveyed non-industrialized cohorts. Maltodextrin, a starch derivative, is used extensively as an emulsifier in processed foods^[8]^. Thus, this hypothesis-generating observation raises the possibility, but does not directly test, that recent changes in diet such as the inclusion of processed foods select for microbial genotypes optimized for metabolism of modern food additives. Perhaps more likely is the increased proportion of simple sugars and dietary starches relative to other dietary fibers in modern Western diets that have driven keystone starch degraders to further specialize and optimize starch oligosaccharide uptake to compete^[9,10]^. However, if supported experimentally with a controlled dietary intervention model, this finding could constitute a particularly vivid example of rapid host-environmental change reshaping microbial evolution in real time.

Industrialized populations appeared to share more sweeps with each other than with non-industrialized populations, implying convergent selection pressures associated with urbanization, diet, and shared lifestyle factors. This observation supports an emerging view that host diet, and in particular carbohydrate nutritional content, act at the gene level to shape not only microbial community composition but also the evolutionary genomic trajectories of individual species. The remodeling of bacterial genomes and communities in recent decades has mirrored the industrialization of society^[11]^. While changes in sanitation, healthcare practices, human behavior, and migration have resulted in microbiome alterations^[12,13]^, the modernization of human diet (typically exemplified as a Western diet) is often highlighted as the major driver of change. This is also reflected by the broad rise of antibiotic resistance genes across diverse microbiomes with elevated use of antibiotics in human medicine and agricultural practice. Western diets high in simple sugars and starches, low in dietary fibers, and replete with food additives and emulsifiers^[8,14,15]^ have provided strong selective pressures that impact microbiome diversity, composition, and function^[9]^. The diversity of carbohydrate-active enzymes (CAZymes) in Western populations is therefore reduced relative to pre-industrialized populations, reflecting bacterial adaptation to the decrease in dietary fibers^[16]^.

Wolff and Garud bolster previous findings that human diets are a major driver of altered microbiome composition in industrialized societies through their evolutionary prism that propels HGT as a centerpiece of bacterial adaptation^[5]^. However, other evolutionary mechanisms such as negative frequency-dependent selection^[17]^, polygenic adaptation^[18]^, and epigenetics may be prominent but are unnoticed by this approach^[19]^. Modeling a gene’s frequency in a given population, its interaction with distant genes, and its expression is difficult to ascertain from bacterial metagenomes alone. Additionally, the dependence on accurate haplotype reconstruction from metagenomic data, reduced sensitivity in low-abundance taxa, or loci exhibiting structural variation rather than SNP-based divergence may further limit the scope of discovery by this approach.

Nonetheless, this work is impactful and portrays the microbiome as an evolving extension of the host where strain- and gene-level variations significantly impact function. The study offers a roadmap to discover connections between microbiome genetics and potential host environmental exposures. Future work involving computational models of microbial metabolism and evolution or the assessment of a genetic variant’s contribution to relative fitness can validate the relevance of gene-specific sweeps as identified by iLDS to microbial adaptation. The altered capacity for carbohydrate degradation and utilization in the microbiome observed here could eventually be revealed to negatively impact human health or underlie chronic disease, though these relationships were not investigated in this study^[11,20]^. In contrast, identifying what bacterial genes are undergoing positive selection may prove to be a critical piece of evolutionary-informed bacterial therapy selection and rational design. As the field moves toward precision medicine, the selective landscape of the recipient microbiome may need to be considered in conjunction with the therapy itself in order to enable better clinical success and address human health.

## DECLARATIONS

### Copyright

© The Author(s) 2026.
